# The American Dental Association

**Published:** 1873-09

**Authors:** D. C. Hawxhurst


### THE AMERICAN DENTAL ASSOCIATION.

BY D. C. HAWXHURST.

The American Dental Association has recently concluded its sessions, and its action may be held to be a fair subject for public criticism. Before proceeding to point out its faults or commend its many merits, I may say that I write in some haste, and with no other expectation than that of fairly laying open to discussion a subject that has needed light let in upon it. There are some who would be glad to see this body hold its place as the very first dental organization in the world—first in its contributions to science, first in the thoroughness and excellence of its discussions, first in the lofty professional attainments of its members, and first in the wisdom and discretion of its legislation for local societies. Many of its friends do now consider it to be, with all its faults, the "highest existing dental authority on this planet;" from which it must of course follow, that no small honor is conferred upon a practitioner when he is made one of its members.

But, how does it happen that this august body, which is supposed not to have its equal at any point nearer to North America, than Saturn or Jupiter, or, possibly not within the limits of the solar system, how does it happen that it has no qualifications for membership? It certainly seems a little singular that a scientific body that aspires to legislate for the dental profession, should be so careless as regards the qualifications of its legislators. If it be a fact that any body who can present a certificate that he has been elected a delegate by his local society, and who can raise the simple fee of five dollars, may at once, and thus easily, become a member of this association, then how are we to maintain for it any high standard of professional acquirement?

For, do we not well know how these delegates are made? They are not always, as might be supposed, selected because they of all others, are best fitted to represent their respective societies. They are not always chosen because of their generous acquaintance with science, or their shining professional qualifications. They are in too many instances elected delegates for the simple reason, that they are willing to go. The society has perhaps already elected its three or four able members, who always go, and is entitled to one or two more delegates. Who can it get to fill these vacant places, and secure for it a full representation ? Probably some very rusty rubber-plate man, who has been let into the society because the treasury was very much in need of his membership fee, is presented and elected. Perhaps it is some young member, who has been admitted to membership on a promise of "reading up ;" or some middle-aged practitioner, old in dentistry and experienced in ignorance, who comes in on the strength of long practice. Or, perhaps this particular local society has no qualifications for admission, but takes in all paying applicants, in which case any of the delegates are liable to be, not only poorly qualified, but not qualified at all. In many cases, all the best members of the local societies, have become permanent members of the National Association, and the whole number of delegates is made up of material not the best—often the poorest.

Indeed, from my observation in local societies, I am quite perpared to maintain that the way into the American Dental Association is a free one, that may be troubled by the illiterate, unscientific, unprofessional, irresponsible quack. If not many men of this lowest grade enter, it is not because of any protecting regulations made by the association, but the rather because of their lack of interest in its labors.

But while the qualifications for admission to this great delegated body stand at zero, some of those societies from which it receives delegates are very rigorous in their examination of candidates for membership. Were they all so, then, indeed, there were no need of this article ; the American Dental Association could then trust in a measure to the local societies, to keep up the high character of its membership. But unfortunately the examples of a high qualification for membership among local organizations are exceptional. May not the incompetent practitioner, who is refused by one society, be accepted by another ? And may he not become their delegate ? If he be ambitious , he is now on the high road to a fulfillment of his dreams. He will soon be a member of the most formidable and scientific dental organization in the world. What need of any further study now? Standing on the same floor, is he not the equal of Taft, and Palmer, and Watt, and McQuillen and the rest?

It seems to me that this matter needs overhauling. Some moderate qualification for admission to membership would act most favorably, both on the local societies, and on the general association.

1—A careful examination of candidates for admission to the American Dental Association, would purify its membership and elevate its general character and tone.

2—The adoption of such examinations would react favorably upon the members of local societies, by stimulating their members to qualify themselves for delegates.

Reserving several other suggestions for future writing, I dismiss this topic with a single remark. It is that I know of no important scientific body in the world, that has long held the respect and confidence of cultivated circles, that did not exercise a wise discretion in the selection of its members.

If this and other important matters shall not receive the attention of those most interested, I greatly fear that those damaging criticisms which have already been made on our National Association, will continue. Already it is to be observed that many of our best members fail to present themselves at its sessions.

